# Analyses and treatments of postoperative nasal complications after endonasal transsphenoidal resection of pituitary neoplasms

**DOI:** 10.1097/MD.0000000000006614

**Published:** 2017-04-14

**Authors:** You Cheng, Fei Xue, Tian-You Wang, Jun-Feng Ji, Wei Chen, Zhi-Yi Wang, Li Xu, Chun-Hua Hang, Xin-Feng Liu

**Affiliations:** a Department of Otolaryngology-Head and Neck Surgery; b Department of Neurosurgery; c Department of Neurology, Jinling Hospital, Nanjing, Jiangsu, China.

**Keywords:** complications, endonasal transsphenoidal resection, nasal, pituitary neoplasms

## Abstract

In this study, we analyze and discuss the treatments of postoperative nasal complications after endonasal transsphenoidal resection of pituitary neoplasms (PNs). We performed 129 endonasal transsphenoidal resections of PNs and analyzed and treated cases with nasal complications. After endonasal transsphenoidal resection of PNs, there were 26 cases of postoperative nasal complications (20.1%), including nasal hemorrhage (4.8%), cerebrospinal fluid rhinorrhea (6.9%), sphenoid sinusitis (2.3%), atrophic rhinitis (1.6%), olfactory disorder (1.6%), perforation of nasal septum (0.8%), and nasal adhesion (2.3%). All patients clinically recovered after therapy, which included treatment of the cavity through nasal endoscopy, intranasal corticosteroids, and nasal irrigation. We propose that regular nasal endoscopic review, specific nasal medications, and regular nasal irrigation can effectively clear nasal mucosal hyperemia-induced edema and nasal/nasoantral secretions, as well as promote regeneration of nasal mucosa, prevent nasal adhesion, maintain the sinus cavity drainage, and accelerate the recovery of the physiological function of the paranasal sinus. Timely treatment of patients with nasal complications after endonasal transsphenoidal resections of PNs could greatly relieve the clinical symptoms. Nasal cleaning is very beneficial to patients after surgery recovery.

## 1 Introduction

Pituitary adenoma is a benign tumor that occurs in the endocrine center pituitary gland. The incidence of pituitary adenoma is about 1 to 7 per 100,000, accounting for about 10% to 15% of intracranial tumors.^[1]^ In recent years, with the development of imaging and examination technologies, the early diagnosis rate of pituitary adenoma has obviously improved.^[2]^ Methods of treatment for pituitary adenomas include drug control, surgery, and radiation therapy. Surgical resection is the first choice for radical pituitary adenoma.^[3]^

In 1907, Schloffer first performed pituitary tumor resection by nasal transsphenoidal approach.^[4]^ In 1962, Hardy further modified this into a standard procedure for microsurgical resection of pituitary adenoma via the oral nasal sphenoid sinus.^[5]^ In 1987, Grifith and Veerapen improved the transsphenoidal pituitary adenoma resection technique. The approach has less trauma and fewer postoperative complications, so it can quickly promote recovery.^[6]^ Transnasal endoscopic resection of pituitary adenomas was first reported in 1992 by Jankowski et al Shikani, Kelly, and Gamea further improved the technique.^[7]^

Endonasal transsphenoidal resection of pituitary neoplasms (PNs) is widely accepted as a safe operation method providing good results. Meanwhile, many complications have been reported in this method.^[8]^ Among common complications are not only nasal hemorrhage and rhinorrhea, but also sphenoid sinusitis, atrophic rhinitis, olfactory disorder, perforation of nasal septum, and nasal adhesion. However, reports of the analyses and treatments of the complications have not been documented. We performed 129 endonasal transsphenoidal resections of PNs in neurosurgery of our hospital from January 2009 to December 2012. In 26 cases, postoperative nasal complications after endonasal transsphenoidal resections of PNs (20.1%) were identified and all of them were treated clinically.

## 2 Methods

### 2.1 Patient summary

We analyzed 129 cases of PNs, in which reliable clinical data were collected. The study sample consisted of 52 men and 77 women, with an average age of 37 years (range: 10–76 years). The disease lasted for on average of 2.6 years (range: 10 days–12 years).

### 2.2 Imaging examination

Preoperative routine magnetic resonance imaging examination was performed. There were 87 cases of microadenoma (maximum diameter <1 cm), 37 cases of large adenoma (maximum diameter 1–4 cm), and 5 cases of giant adenoma (maximum diameter >4 cm).

### 2.3 Surgery details

In all 129 cases, endonasal transsphenoidal resections of PNs were performed. Each person signed the operation informed consent before surgery. Because there was no harm to the patient, the ethical approval was not necessary. Among these cases, 95 were operated using a microscope and 34 using a neuroendoscope. Furthermore, 31 tumors had indurated texture and 17 were highly invasive; 12 patients had tumor-induced stroke symptoms, and in 9 cases, patients experienced postoperative recurrence and returned to our hospital for a second surgery.

### 2.4 Tumor types

According to endocrinological and pathological examinations, there were nonfunctioning adenomas in 37 cases, prolactin growth hormone adenomas in 42 cases, growth hormone-secreting adenomas in 10 cases, cortical hormone adrenal adenomas in 31 cases, and 9 cases of other types.

### 2.5 Surgery results

Among all cases, 117 cases (90.7%) were of total resection and 12 cases (9.3%) were of subtotal resection. These patients were followed up for 7 to 55 months, with an average of 19 ± 7.2 months.

### 2.6 Analyses of postoperative nasal complications

**Figure 1:**
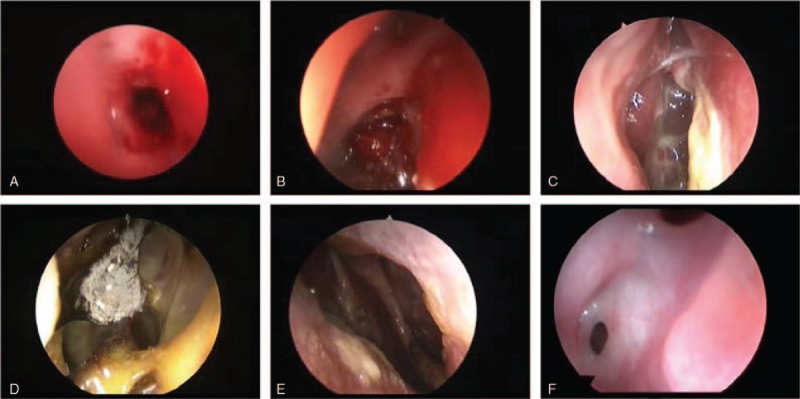
Nasal endoscopy results. (A) Nasal hemorrhage; (B) cerebrospinal fluid rhinorrhea; (C) sphenoid sinusitis (with sphenoid sinus polyp); (D) atrophic rhinitis (with fungal infection); (E) nasal endoscopy showing nasal septum perforation; (F) right nasal adhesion.

Analyses of postoperative nasal complications after endonasal transsphenoidal resections of PNs are shown in Table 1. The results of nasal endoscopy are shown in Figure 1.

**Table 1:**
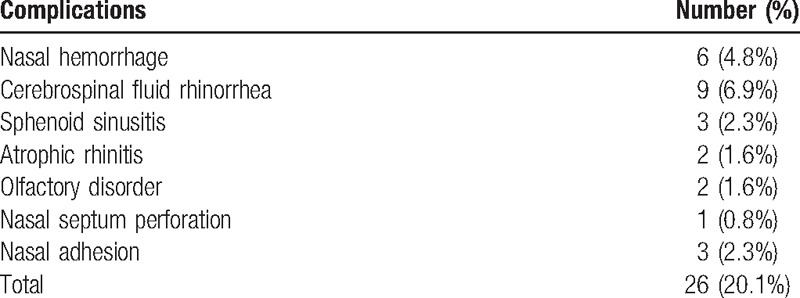
Postoperative complications in nasal cavity.

### 2.7 Treatments and results of postoperative nasal complications

#### 2.7.1 Nasal hemorrhage

**Figure 2:**
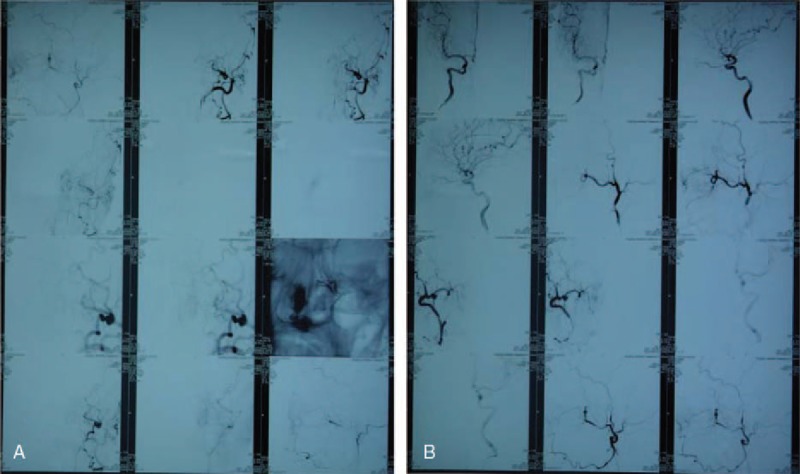
Patients with intractable nasal bleeding underwent DSA examination 1 month after operation: (A) before vascular embolism; (B) after vascular embolism.

There were 6 cases of postoperative nasal hemorrhage (4.8% of all cases). One case of refractory nasal hemorrhage presented as a repeated and vast right nostril hemorrhage 1 month after neuroendoscopic surgery. The case was confirmed as maxillary artery aneurysm using digital subtraction angiography (DSA) and was treated by endovascular embolization treatment (EVET) (Fig. 2). Three cases of nasal mucosa with diffuse blood oozing were initially treated with inflation sponge to stop the bleeding; 48 hours later, the sponge was replaced with a treatment with compound menthol nasal drops. Two cases of traumatic hemorrhage of sphenopalatine artery were treated with electrocoagulation hemostasis with the nasal endoscope. All 6 cases of nasal hemorrhage were cured after the treatments.

#### 2.7.2 Cerebrospinal fluid rhinorrhea

Among 9 cases (6.9% of all cases) of postoperative cerebrospinal fluid rhinorrhea, one 1 with mild symptoms stopped on its own, 6 cases stopped after continuous lumbar cisterna drainage, and 2 cases were cured by surgical repair.

#### 2.7.3 Sphenoid sinusitis

**Figure 3:**
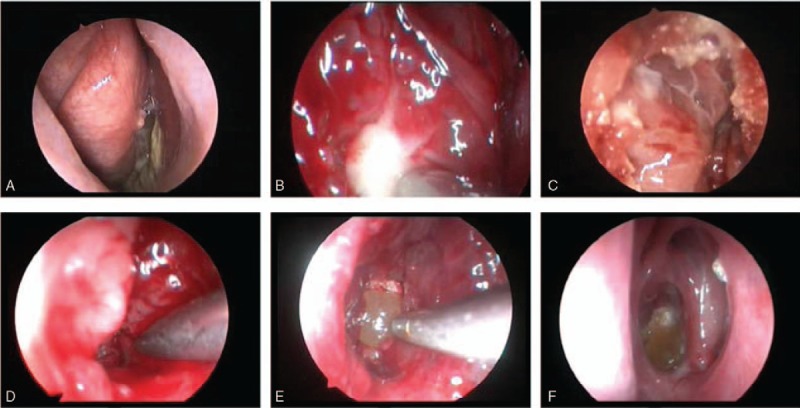
Endoscopic treatment of a male patient with postoperative sphenoid sinusitis after resection of the nasal sella area tumor. (A) Endoscopic image before treatment, characterized by stenosis of the right aperture of the sphenoidal sinus, swollen nasal mucosa, polypoid change, and purulent secretion of the opening of the sphenoidal sinus. (B) Image during the treatment, showing submucosal abscess. (C) Image taken 1 month after the treatment, showing significant swelling of the sphenoid sinus cavity mucosa. (D) Image taken 2 months after endoscopic treatment, where the opening of the sphenoidal sinus can be seen and local drainage is good. (E) Image taken 4 months after the treatment, where the opening of the sphenoidal sinus is good, the swollen mucosa of the sphenoid sinus cavity is no longer apparent, and epithelization is appearing on parts of the mucosa. (F) Final image taken 8 months after endoscopic treatment, showing good opening of the sphenoidal sinus and epithelized mucosa; some residual ointment for reduction of the inflammation is also visible.

**Figure 4:**
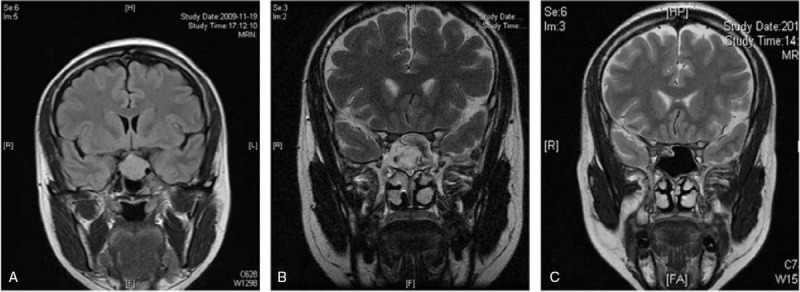
Coronal magnetic resonance imaging of the head of a female patient, who underwent resection of the transsphenoidal pituitary adenomas (A) before surgery; (B) 3 months after surgery and before treatment; (C) 47 months after surgery and 44 months after treatment.

**Figure 5:**
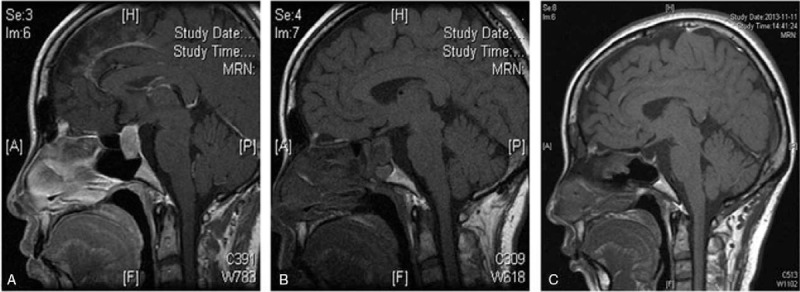
Sagittal magnetic resonance imaging of the head of the female patient in Figure 4. 4.(A) before surgery; (B) 3 months after surgery and before treatment; (C) 47 months after surgery and 44 months after treatment.

Three cases of postoperative sphenoid sinusitis (2.3% of all cases) were cured by regular nasal endoscopic cavity cleaning, intranasal corticosteroids, and nasal irrigation. (Figs. 3–5)

#### 2.7.4 Atrophic rhinitis

**Figure 6:**
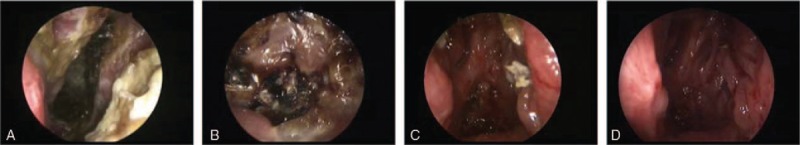
Case of atrophic rhinitis treated by nasal endoscopy: (A) before treatment; (B) 2 weeks after treatment; (C) 4 weeks after treatment; (D) 16 weeks after treatment.

Regular nasal endoscopic cavity cleaning, compound menthol nasal drops, and nasal irrigation markedly improved the conditions of 2 patients with postoperative atrophic rhinitis (1.6% of all cases) (Fig. 6).

#### 2.7.5 Olfactory disorder

Two cases of postoperative olfactory disorder (1.6% of all cases) were treated with endoscopic breakdown of the adhesion, oral, and nasal corticosteroids, nerve nutrition, and nasal irrigation for 3 months. The condition of one patient was improved, but the treatment had no effect on the other case.

#### 2.7.6 Perforation of nasal septum

One case of postoperative perforation of nasal septum (0.8% of all cases) was treated with endoscopic repair operation. In situ restoration was performed using mucoperiosteal pedicle flap of the floor of the nasal cavity. In the postoperative follow-up, the perforation was healing well.

#### 2.7.7 Nasal adhesion

Three cases of postoperative nasal adhesion (2.3% of all cases) were treated with endoscopy-assisted low-temperature radiofrequency ablation to isolate the adhesion, nasal isolation by NasoPore, nasal corticosteroids, nasal irrigation, and additional standard treatment. During 6-month follow-up, no recurrence or new adhesions were observed.

## 3 Discussion

### 3.1 Nasal hemorrhage

The rate of occurrence of internal carotid artery (ICA) injury in endonasal transsphenoidal resection of PNs is 1%; the probable causes include intraoperative deviation from the center line, injury during resection of the tumor invading the cavernous sinus, and accidental injury when removing the sphenoid sinus mucosa. Prevention of the hemorrhage could be facilitated by a median approach and good knowledge of the sellar and surrounding regions. In this group, there was 1 case of repeated and vast right nostril hemorrhage 1 month after neuroendoscopic surgery, which was confirmed as left maxillary artery aneurysm with DSA. This case was cured with EVET. This complication may have been related to vascular injury, but it is impossible to infer the exact mechanism retrospectively.

Among the other 5 cases of nasal hemorrhage, there were 3 cases of nasal mucosa with diffuse blood oozing and 2 cases of traumatic hemorrhage of the sphenopalatine artery. To prevent nasal hemorrhage, the following points should be considered: taking into account the sphenopalatine artery and its branches distributed in the mucosa of the cut position, electrocoagulation should be performed, when the mucosa is being cut and to decrease the occurrence of delayed hemorrhage, nasal irrigation and use of compound menthol nasal drops are necessary.^[9]^

### 3.2 Cerebrospinal fluid rhinorrhea

The rate of occurrence of cerebrospinal fluid rhinorrhea is around 0.6% to 5.3%.^[10]^ Most cases are caused by the opening of the suprasellar cistern, falling of the suprasellar arachnoid into the sellar through expanded sellar septal foramen resulting in an accidental burst injury, a cut to the front of the floor of the sella turcica allowing for the arachnoid to be easily cut open when the tumor is too small, or the destruction of the surrounding tissue structure by the tumor. Once an intraoperative cerebrospinal fluid leakage has been found, repair should be performed strictly during the operation. In all cases in this group, “sandwich treatment,” that is, gelatin sponge and biomedical fibrin glue to pack and seal the inner layer and the floor of the sella turcica and bone fragments and ear-brain glue to reinforce the outer layer, was adopted.

Based on our experience, we would like to provide some suggestions on surgical repair of cerebrospinal fluid rhinorrhea. First, once the position of the hole is determined, granulation tissue should be scraped clear before repair and the surgeon should enlarge the site around the tear by about 2 mm to expose the bone wall and make fresh wound to help heal the hole. Second, careful choice of the repair materials should be considered and the tear should be treated cautiously. If the hole is large, temporal fascia or temporal fascia with nasal septal cartilage and middle turbinate bone may be appropriate repair materials, whereas if the hole is small, temporal fascia or temporal fascia with albumin glue may be a better choice. Third, NasoPore and expanding sponge should be adopted to decrease the stimulation of the nasal mucosa, relieve edema, improve patients’ comfort, and support the transplant.

For cases of sustained cerebrospinal fluid rhinorrhea, the tear can be precisely located by examining with a nasal endoscope and imaging techniques. It is a method for treatment of minor injuries and can be better for some patients, who were unresponsive to the typical treatment.^[11]^

The 9 cases of cerebrospinal fluid rhinorrhea in this group accounted for 6.9% of all cases, which was higher than the occurrence rate reported in the literature. Our research suggested that the second surgery, intraoperative cerebrospinal fluid rhinorrhea, and surgeon's experience may possibly play a role in the occurrence of cerebrospinal fluid rhinorrhea and should be taken into account during the preoperative risk assessment. Early detection and prevention, with positive and decisive management, has to be performed for high-risk patients.^[12]^

### 3.3 Sphenoid sinusitis

Postoperative sphenoid sinusitis after endonasal transsphenoidal resection of PNs is easy to diagnose based on the standards for nasosinusitis proposed in EPOS-2012.^[13]^ There were 3 cases of postoperative sphenoid sinusitis (2.3% of all cases) in this group. Batra et al's^[14]^ study reported 200 cases of endonasal transsphenoidal resection of PNs. They found that the occurrence rate of postoperative nasosinusitis was 7.5% and the average duration was 2.9 years.

The main causes of postoperative sphenoid sinusitis after endonasal transsphenoidal resection of PNs are: ignoring the nasal cavity and sinus functions, local nasal treatments, and drug applications during the perioperative period; nasal mucosa, especially mucosa of the sphenoid sinus, being heavily injured during operation and the drainage of the sphenoid sinus being obstructed because of the damage to the mucosa mucus-cilium system^[15]^; And ignoring the regular postoperative endoscopic inspection, leading to failure of fluid drainage of the sphenoid sinus.

During the endonasal transsphenoidal resection of PNs, surgeons should adopt an appropriate surgical method, combining the examination of CT and endoscopy images, and focusing on the 3 points. First, they should make sure that the middle turbinate is retained; the lateral nasal wall should be adopted for a direct surgical approach when the patient has deviated nasal septum.^[16]^ Second, the surgeons should pay attention to the conservation of the nasal mucosa. Third, the postoperative nasal packing should be performed precisely and gently; the middle turbinate should be fixed at the normal position, and the packing should be removed within 48 hours.

The nasal and nasoantral perioperative treatment of the endonasal transsphenoidal resection of the PNs has not been considered enough by most neurosurgeons; therefore, postoperative endoscopic observation and treatment have not yet become part of the routine procedure. Otorhinolaryngologists are at an advantage in the adoption of the nasal endoscope to observe and treat sphenoid sinus diseases precisely and systematically, and their expertise could prove highly useful during the surgery. The strengths of the nasal endoscope to treat sphenoid sinus diseases in otolaryngology surgery are^[17]^ accurate localization, multidirectional, multiview observation of the region and the extent of sphenoid sinus disease, the ability of regular review and treatment under nasal endoscope to establish sufficient drainage pathway, and protection of nasal physiological functions, mild injury, and postoperative management taking into account the concept of functional surgery.

Based on our research, close follow-up and review, according to the condition of the nasal endoscopic sphenoid sinus cavity, and the local application of medicines, such as glucocorticoids, are optimal methods to prevent and treat postoperative sphenoid sinusitis after the endonasal transsphenoidal resection of PNs.

### 3.4 Atrophic rhinitis

There are 3 causes of postoperative atrophic rhinitis after endonasal transsphenoidal resection of PNs: if the disease is induced intraoperatively, the normal structure of the nasal-paranasal sinus mucosa of the nasal cavity would have been damaged; intraoperative and postoperative nasal hemorrhage leading to more and deeper electrocoagulation of the nasal mucosa and cavity; and use of very tight postoperative nasal packing and leaving it in place for too long. For the issues mentioned above, we suggest protecting the prenaris and limen nasi, protecting the nasal mucosa, and packing the nasal cavity accurately.

Although Jho^[18]^ considered the postoperative packing of nasal cavity not necessary, based on our experience, we still find it essential for the restoration of mucosa, hemostasis by compression, and fixing of the treatment materials to the floor of the sella turcica.

Regular postoperative examination and treatment should be performed until epithelization. For the preservation of normal growing epithelium, continuous clearing of nasal scabs, vesicles, small polyps, and fibrous adhesions is essential. Surgeons should avoid disturbing the reconstruction materials on the floor of the sella turcica during the postoperative treatment performed with a nasal endoscope 3 months later so that surgical cavities can completely recover. The condition of these patients would be markedly improved with regular nasal endoscopic cavity cleaning (for patients without cerebrospinal fluid rhinorrhea), compound menthol nasal drops, and nasal irrigation. The recovery of nasal postoperative mucosa could be also promoted by endoscopic cavity cleaning. Furthermore, cleaning the nasal cavity with normal saline after the application of compound menthol nasal drops facilitates sinus mouth drainage, elimination of mucosal edema, prevention of nasal adhesion, and recovery of the nasal mucosa.

### 3.5 Olfactory disorder

Olfactory disorder is another postoperative nasal complication after endonasal transsphenoidal resection of PNs.^[19]^ It is often caused by the placement of the nasal dilator, which injures and compresses the nasal mucosa, contributing to ischemia, excessive burning of the hemorrhagic mucosa, and ineffective protection of nasal mucosa, especially by the injury of turbinal and septal mucosa, which consist of olfactory nerve endings. Two cases in this group (1.6% of all patients) had different degrees of olfactory disorder, which was confirmed by a postoperative review. At the same time, other researchers found the occurrence of olfactory disorder to be 9% and 10.4%.^[20,21]^

During the operation, surgeons should effectively protect the nasal mucosa, especially from injury of the turbinal and septal mucosa, which consist of olfactory nerve endings. It is safe to operate at the range of 10 mm from the cribriform plate beside the nasal septum and 15 mm from the cribriform plate beside the turbinate.

After endonasal transsphenoidal resection of PNs, to control nasal mucosa inflammation and avoid olfactory disorder, subjective and objective examination should be emphasized, as regular review of the nasal cavity. The objective of the follow-up is to prevent complications, like the spread of inflammation, local infection, nasal adhesion, and blockage of the sinus drainage channel. The cleansing of edematous mucosa, blood scabs, granulation, and vesicles can improve the condition of nasal-paranasal sinus ventilation and drainage, thus promoting the recovery and olfactory function. Moreover, for the recovery of olfactory function, it is necessary to perform comprehensive treatments such as endoscopy, oral and nasal corticosteroids, nerve nutrition, and nasal irrigation. The 2 cases of olfactory disorder in this group were treated as mentioned above for 3 months. The condition of 1 patient improved, but the treatment had no effect on the condition of the other patient.

### 3.6 Perforation of the nasal septum

Perforation of the nasal septum can result in symptoms such as nasal obstruction, headache, dryness of the nostrils, nasal hemorrhage, and whistling sounds. If the perforation is more to the front of the septum, the clinical symptoms will become more severe.^[22]^ Patients with perforation often need operation to recover the normal respiratory airflow of the nasal cavity and to eliminate clinical symptoms.

Most of the repair of the nasal septal perforation can be performed under a nasal endoscope. Presutti et al^[23]^ suggested that a nasal endoscope can provide abundant room for the separation and suture of the mucosal flap and the full exposure of the visual operative field. Considering the extensive range of repair materials, the choice of resources should be adequately aligned to the surgeons experience and operative skills. The common operative repair methods, among others, are free flap reconstruction, relieving tension composite flap suture, repair with composite flap, repair with inferior nasal concha pedicle flap, and repair with the mucoperiosteal pedicle flap of the floor of nasal cavity.

A single case in this group was treated with reconstruction operation of the nasal septal perforation. In situ restoration was performed by adopting the mucoperiosteal pedicle flap of the floor of the nasal cavity. In the postoperative follow-up, the perforation was healing well. In our experience, the endoscopic operation of the nasal septal perforation has the following features: clear view and straightforward procedure; the mucoperiosteal pedicle flap of the floor of the nasal cavity can provide blood supply, and the transplants survive and grow easily. There should be a 2-mm overlap between the restoration and tissue surrounding the perforation; precise suture and fixing of implants should be performed, and there should be no strain in the suture after the transplant. During postoperative care, one should avoid disturbing the transplants and use normal saline to maintain the level of moisture of the nasal cavity.

### 3.7 Nasal adhesion

Postoperative nasal adhesion may be attributed to rhinostenosis, mucosal edema, postoperative drifting of the middle nasal concha, hyperplasia of the granulation tissue, dysfunction of the mucosal epithelium, cicatricial contracture, lack of timely and proper follow-up, and nasal endoscopic review. After regular cleaning and separation of nasal adhesion, reinstatement of inflammation of nasal mucosa and injury healing would inevitably lead to new granulation tissue and scaring.^[24]^ Therefore, simple nasal adhesion separation can easily lead to new adhesion. Wound healing is a course of competition between mucosal epithelium development and lesions regeneration, which is likely to continue for 6 months.

Reduction of unnecessary damage as soon as possible, and gentle, precise operation to protect the nasal cavity, naso-antral normal mucosa, and regenerating tissues, can prevent nasal adhesion and lead to rapid cavity epithelization.

Three cases of postoperative nasal adhesion (2.3%) were treated with endoscopy-assisted low-temperature radiofrequency ablation to isolate the adhesion, nasal isolation by NasoPore, concha nasalis media reconditioning, removing of abnormal nasal cavity structure, nasal corticosteroids, nasal irrigation, and follow-up standard treatment. No recurrence or new adhesions was observed during the 6-month follow-up. The treatment and outcomes illustrated that perioperative application of nasal corticosteroids, precise reconditioning of concha nasalis media, reduction of mucosal injury, regular postoperative cavity clearing, and timely treatment of adhesion can lower the occurrence of postoperative nasal adhesion.

### 3.8 Internal carotid artery injury

After transsphenoidal surgery, the incidence rate of ICA injury was 1%, owing to intraoperative midline shift, resection of tumor invading the cavernous sinus, and sphenoid sinus mucosa damage removal injury. Raymond et al^[25]^ reported 1800 cases of transsphenoidal surgery. Severe carotid hemorrhage occurred in 21 cases, injury of ICA in 17 cases, palatal artery injury in 4 cases, and death in 3 cases. Surgical approach along the midline, familiarity with the sellar region, and surrounding anatomy are keys to prevent ICA injury. The incidence of ICA injury in transsphenoidal surgery was 1% and fatality rate was about 14%.^[26]^ Methods for prevention and treatment of intraoperative carotid hemorrhage are as follows: according to the preoperative imaging data, a detailed analysis of the boundaries of the cavernous sinus and cavernous segment of the ICA and tumor should be performed; carotid pressure, aspirator, and gelatin filling in the lateral cavernous sinus should be immediately applied; the ability of judgment on endoscopic anatomy and pathological anatomy should be improved.^[27]^

## 4 Conclusions

We propose that regular nasal endoscopic review, specific nasal medications (intranasal corticosteroids, compound menthol nasal drops, cefuroxime nasal spray), and regular nasal irrigation (in patients without cerebrospinal fluid rhinorrhea) can effectively clear nasal mucosal hyperemia edema and nasal/naso-antral secretions, as well as promote regeneration of lesions, prevent nasal adhesion, maintain the sinus cavity drainage, and accelerate the recovery of the physiological function of the paranasal sinus. The treatments listed above are an effective choice for reduction of postoperative nasal complications after endonasal transsphenoidal resection of PNs.^[28]^ The professional treatments of postoperative nasal complications can effectively improve the nasal cavity/nasoantral function and decrease clinical symptoms, so as to improve the patients’ quality of life.
